# Exploring nursing managers’ perceptions of nursing workforce management during the outbreak of COVID-19: a content analysis study

**DOI:** 10.1186/s12912-021-00546-x

**Published:** 2021-01-29

**Authors:** Sarieh Poortaghi, Mehraban Shahmari, Akram Ghobadi

**Affiliations:** 1 PhD of Nursing Education, Tehran University of Medical Sciences, School of Nursing and Midwifery, Community Health Nursing Department, Tehran, Iran; 2 School of Nursing and Midwifery, Tehran University of Medical Science, Tehran, Iran; 3 PhD student in Nursing, School of Nursing and Midwifery, Tehran University of Medical Science; Lecturer, School of Nursing and Midwifery, Kermanshah University of Medical Sciences, Kermanshah, Iran

**Keywords:** Nursing, Management, COVID-19, Qualitative research, Workforce recruitment

## Abstract

**Background:**

The COVID-19 pandemic is a serious threat to public health worldwide. Therefore, a coordinated global response is needed to prepare health care systems to face this unprecedented challenge. Proper human resource management can increase nurses’ productivity and quality of care. Therefore, the present study aims to explore the nursing managers’ perception of nursing workforce management during the outbreak of COVID-19.

**Methods:**

This is a qualitative study with conventional content analysis using Granheim and Landman approach. In this study, 15 nursing managers were selected by purposeful sampling method. Data were collected using in-depth semi-structured interviews. Ethical considerations were applied to all stages of the study. In this study, MAXQDA software version 10 was used to help manage the data.

**Results:**

66% of the participants (10/5) were female. The mean age of participants was 44 years, mean work experience of 19 years, and mean management experience of 9 years. Three categories and seven sub-categories emerged from the data analysis: 1) management of workforce recruitment (volunteer workforces, non-volunteer workforces), 2) management of workforce arrangement (flexible work schedule, rearrangement of the workforce), and 3) management of workforce retention (preventive measures, motivational measures, and psychological support).

**Conclusion:**

Management in critical situations requires the use of flexible and situational management principles to recruit, arrange and retain workforce, and also to compensate for the lack of manpower.

**Supplementary Information:**

The online version contains supplementary material available at 10.1186/s12912-021-00546-x.

## Background

The novel coronavirus pandemic started in Wuhan, China, in December 2019 and has spread dramatically around the world since then, despite quarantine and containment measures [1–4]. This new viral disease is the third most common viral disease in the twenty-first century after Severe Acute Respiratory Syndrome (SARS) and Middle East Respiratory Syndrome (MERS) [5, 6]. According to the World Health Organization (WHO), as of September 26, 2020, about 213 countries all around the world have been affected by this disease, resulting in 32,700,000 confirmed cases and more than 993,000 deaths. In Iran, as the 13th country affected by this disease, the number of cases on the same date has reached more than 439,000 cases and the number of deaths has reached more than 25,000, which is unprecedented in the history of Iran, and of course, this statistic is changing every day [7]. Coronavirus Disease 2019 (COVID-19) pandemic has placed a heavy burden on the health care system, particularly on nurses who are faced with the greatest challenges regarding the unprecedented outbreak of coronavirus worldwide [8]. COVID-19 has created unprecedented professional, social, and psychological challenges for nurses [9–11]. By recognizing the sources of health care workers’ concerns, health care managers and leaders can provide targeted approaches to address these concerns and provide specific support to their health care workforce. This study can guide nursing managers in this regard [12, 13]. Therefore, a coordinated global response is needed to prepare health care systems to face these challenges [14]. The World Bank (2017) emphasizes that global readiness for pandemic is crucial for global security and should be considered as part of a program for strengthening health care systems [15]. Managers must be prepared to respond to the effects of this pandemic on nurses [16]. Even with advances in healthcare and virus control technology, real success is not possible without effective leadership. In order to prepare hospitals to deal with epidemics, the WHO (2014) recommends that staff shortages should be anticipated due to absenteeism and increased demand for services, and a plan should be put in place to address this shortage, including the use of additional personnel [13, 17]. Low nursing staffing levels, particularly nurse-patient ratios, are themselves associated with the spread of pathogens in health care settings and the risk of the outbreak [18, 19]. Proper staffing and planning is definitely one of the main tasks of hospitals. The workforce recruitment method is a regular process based on a logic that is used to determine the correct number and type of manpower needed to provide standard care in a health care institution [20]. Appropriate human resources can increase the productivity of nurses and the quality of care, which is the main mission of the health care system [21].

Since this is the first time that an infectious disease has spread to this extent in Iran and it may not be the last, the COVID-19 experience in Iran provides a unique opportunity to study the new pandemic threat to nursing managers in health care systems, which unlike other countries, it is fighting this pandemic despite severe economic sanctions. Therefore, the researchers in this study sought to explore the nursing managers’ perception of workforce management during the COVID-19 pandemic. It is hoped that the findings of this study will provide public health officials and policymakers with useful insights so that, they will be able to manage nursing staff in possible emerging epidemics more appropriately.

## Methods

### The aim, design, and setting of the study

The present study aims to explore the nursing managers’ perception of nursing workforce management during the outbreak of COVID-19. This is a descriptive qualitative study with a conventional content analysis approach using Granheim and Landman method, which was conducted in 2020. The study setting of hospitals was from different cities in Iran. In cases where the interviews were in person, it was in the managers’ room with full observance of the health protocol and prevention of the movement of various people. Also, if there was a possibility that the participants were infected, it was carried out via the telephone.

### Study participants

In this study, 15 nursing managers of various hospitals in different cities of Iran were selected by purposeful sampling. Individuals with various characteristics in terms of age, gender, education, general work experience, managerial work experience, and management levels were selected. Finally, with access to suitable and qualified participants, data were collected and analyzed simultaneously over 3 months period. The inclusion criteria for the study were; willingness to express personal perceptions on how nursing staff should be managed during the outbreak of COVID-19, being a nursing service manager, clinical supervisor, or head nurse in hospitals that offer service to COVID-19 patients, and being involved in human resource planning and management at the time of sampling and the beginning of COVID-19 outbreak.

### Data collection procedures

In order to collect data and access valid and reliable information, a semi-structured and in-depth interview was conducted. The interview began with general questions about demographic characteristics and then, more specific open-ended questions related to the purpose of the study based on the interview guide were asked (Box 1). Interview guide questions were developed based on expert’s opinions. The minimum interview time was 30 min, and the maximum interview time was 60 min.

### Ethical considerations

The proposal of this study was approved by the Ethics Committee of the Research Council of Tehran University of Medical Sciences (TUMS), with the IR code: TUMS. REC. 1399. 442.

The study objectives were first explained to the participants and written informed consent was obtained from those who wished to participate in the study. Permission was also obtained to record the interview with an audio recorder. The participants were reassured about the principles of confidentiality and anonymity, the voluntary entry into the study, and the freedom to withdraw from the study at any time without any consequences. The time and place of the interview were chosen in coordination with the participants.

### Data analysis

In this study, the conventional content analysis method was used to analyze the data. Content analysis is one of the methods of qualitative analysis used to classify the words and phrases in the text. Content analysis is also one of the research methods used to systematically and objectively describe the content obtained from communication [22]. In this study, data analysis was performed using Granheim and Landman’s approach. Granheim and Landman suggested five steps for analyzing the content of qualitative data: 1) Implementing the entire interview immediately after each interview, 2) Reading the entire text several times to get an overall understanding of its content, 3) Determining semantic units and basic codes, 4) Classifying primary codes in more comprehensive categories, and 5) Determining the main theme of categories [23].

The interviews were recorded and implemented by the “Word” software at the earliest opportunity. After implementation, the text of each interview was read several times to get a general understanding of its content. Then, the semantic units were identified according to the study objectives and primary codes were extracted from them. The codes were categorized in terms of similarities and differences, forming main categories and sub-categories, and this process continued until the main categories were extracted. MAXQDA10 software was used to manage data.

### Trustworthiness

In this study, different methods were used to increase the validity of the study. For instance, the data were coded and classified independently by the researchers, and the extracted codes were reviewed by the research team. Then, the categories obtained from the analysis were compared with each other. In case of a disagreement over the categories, the debate continued until an agreement was reached. The member check method was also used so that, the extracted codes were given to some of the participants and they were asked to confirm the extracted codes and express their corrective opinions. Peer review and audit was carried out on data. So, some interviews were randomly selected and given to some researchers who were familiar with the qualitative method but were not part of the study to review the data and announce their suggestions and feedback. To ensure the transformability of the study, the participants with maximum variation were selected.

## Results

66% of the participants (10/5) were female. The mean age of participants was 44 years, mean work experience of 19 years and mean management experience of 9 years. All demographic characteristics of the participants are presented in Table 1.

**Table 1:** The demographic characteristics of nursing managers who participated in the study

Demographic characteristics		Frequency
Gender	male	5 (33.3o %)
	female	10 (66.70)
Marital status	single	1 (6.70%)
	married	14 (93.3.0%)
Education	Bachelor’s degree	6 (40.00%)
	Master’s degree	9 (60.00%)
Management level	head nurse	11 (73.30%)
	clinical supervisor	2 (13.30%)
	nursing service manager	2 (13.30%)
General work experience	Mean ± SD	19.60 ± 4.77
Management experience	Mean ± SD	9.13 ± 7.49
Age	Mean ± SD	44.46 ± 4.40

In this study, nursing managers perceived the management of nursing staff in the COVID-19 crisis very different from other crises and maneuvers. Three categories and seven sub-categories emerged from the data analysis: 1) management of workforce recruitment (volunteer workforces, non-volunteer workforces), 2) management of workforce arrangement (flexible work schedule, rearrangement of the workforce), and 3) management of workforce retention (preventive measures, motivational measures, and psychological support) (Table 2).

**Table 2:** Categories and sub-categories extracted from the data

Main category	Sub-category	Semantic unit
Management of workforce recruitment	Volunteer workforces	calling volunteers from other wards, hospitals, cities, and other provinces, employing non-specialist volunteer workforces
	Non-volunteer workforces	moving nurses between wards, recruiting contract and temporary staff, extension of contractual forces, speeding up the recruitment process for those who passed the selection test
Management of workforce arrangement	Flexible work schedule	relocating pregnant or lactating forces, moving forces with underlying disease, moving forces treated with immunosuppressive drugs or family members, dynamic adjustment of shift work schedules
	Rearrangement of workforce	combine of new and experienced workforces, adjust the number and proportion of the workforce
Management of workforce retention	Preventive measures	provide training in various ways, provide PPE, evaluate and monitor the correct use of PPE, reduce unnecessary exposure, eliminate combined shifts, increase the off-time between shifts, eliminate mandatory overtimes, reduce monthly working hours, separate the spaces, identify COVID nurses in emergency departments, assign codes to patients with suspected respiratory symptoms, nutritional management, pavilion, and restroom management, allocation of a dedicated place for quarantine of affected personnel, officials as a role model in the correct use of PPE.
	Motivational measures	The strong presence of managers in the field, more cooperation with staff, leadership alongside management, close communication between officials and staff, financial incentives, public donations; charities; public and private institutions, presenting the appreciation of public and officials to employees, allocation of working hours coefficient, the promise of leave
	Psychological support	the possibility of relocation for anxious staff, non-compulsory work in the COVID department, providing psychological counseling, playing happy songs in the ward, live concert performance, creating an intimate atmosphere in the ward

### Management of workforce recruitment

As the disease process prolonged, measures were needed by nursing managers to compensate for the shortage of manpower. These measures include two subcategories of recruitment of volunteer and non-volunteer workforces.

#### Volunteer workforces

Recruitment of volunteer workforces included calling volunteers from other wards, other hospitals, other cities, and in some cases other provinces.

*“The management did this coordination. The nurses in wards that did not have any patients were sent to COVID wards as an auxiliary workforce. Some staff volunteered to work in the COVID wards. Participant No. 10.”*

#### Non-volunteer workforces

Recruitment of non-volunteer workforces was in the form of relocating nurses from other wards to assist COVID wards, recruiting and extending the contract and temporary staff, and speeding up the recruitment process for those who passed the selection test. These measures were carried out in coordination with the hospital and provincial nursing management office.

*“Employees who had recruitment test before the COVID-19 outbreak were asked to come to work. In the COVID-19 crisis, because they had a series of administrative paperwork left, they sent them to work as a temporary workforce. Participant No. 3.”*

### Management of workforce arrangement

If the number of staff in the ward was sufficient, changes and displacements had to be made in the layout and arrangement of the workforces. The management of workforce arrangement includes two sub-categories: flexible work schedule, rearrangement of the workforce.

#### Flexible work schedule

It provided an opportunity to move some of the staff to non-COVID wards due to reasons such as; pregnancy, lactation, being treated with immunosuppressive drugs, and the presence of underlying diseases such as MS (Multiple sclerosis), myasthenia gravis, and asthma, or having acute problems such as cancer or chemotherapy in family members.

*“We had nurses who had myasthenia gravis or heart problem. I also told one of my staff that you don’t have to work on this ward if you have an underlying problem and I will transfer you to other wards if you want to. One staff had to go on leave because she had lupus and had to use steroids. Participant No. 3.”*

Due to the uncertainty in the disease process, the temporary increase or decrease in the number of patients and regular opening and closure of the wards, the unpredictability of the situation and therefore planning for it was one of the biggest management problems. By observing the admission statistics on a daily and weekly basis and convening various meetings between the head nurses and the officials, once month plans were made according to the conditions and requirements of the time, and this schedule was reviewed at shorter intervals.

#### Rearrangement of workforce

In parallel with the increase in the number of patients, new wards were established or changed to COVID ward. In the meantime, due to the nature of the disease, which had led to an increase in demand for intensive care beds, there was a shortage of experienced nurses in the intensive care unit (ICU) more than other nurses. There was a need to rearrange staff in terms of the number and proportion of staff required in each shift, which was somewhat solved by moving nurses working in general wards and using them in ICU alongside experienced staff.

*“In a normal ward like the heart surgery ward, normally its staff arrangement is 3 nurses in the morning shift, and 2 in the evening and night shift. But when it was changed to the ICU, we had to allocate 9-10 staff in the evening and night shift. Participant No. 3.”*

Due to the arrival of new and temporary staff and sometimes some staff at the same time, nursing managers had to train this staff and introduce them to the ward’s routines, and also COVID-19 disease and treatment. They also had to change the arrangement of staff and create a combination of novice staff and experienced ones in each shift to provide better care to patients.

*“Well, in terms of workforce arrangement, in each shift we tried to have a really alert nurse who is familiar with everything and knows how to work with the device so that, the new staff who are a little weaker, wouldn’t have a problem. Participant No. 5.”*

### Management of workforce retention

In addition to measures taken to recruit or change the arrangement of staff, measures were also taken for staff working in COVID wards so that, they could continue to work with less concern, more hope, and more resilience. These measures are expressed in three subcategories: preventive measures, motivational measures, and psychological support.

#### Preventive measures

Nursing managers took measures to reduce the risk of infection and raise the threshold of personnel resistance. One of the measures taken by managers to increase the level of awareness and thus reduce the likelihood of staff infection was to provide training in various ways on the importance, manner, and order of using personal protective equipment (PPE). After the initial training, the supervisors monitored and observed the learners’ behaviors during the shift. In addition to providing, making available, managing, and rationing PPE, the managers were a good role model in terms of how to use it.

Some of these measures involved work shift planning and staff arrangement. During this time, attempts were made to eliminate combined shifts and adjust shorter shifts. The distance between shifts was increased so that, the colleagues could recover, and wider shifts were created so that, they could have more rest. Monthly working hours were reduced by as much as 30% and in addition to reducing the risk of infection, mandatory overtime was eliminated.

*“The training started on the day of the announcement of the Corona outbreak. The task of the supervisors was to provide training to all staff in various ways on how to protect themselves and how to deal with these patients in general. Participant No. 7.”*

*“We also try to provide them with equipment during their shifts. Everything was always available to the staff in all shifts. Participant No. 10.”*

*“We have reduced working hours. For example, if you used to work 150 hours a month, it was reduced to about 100 hours a month, which means about 7 to 8 (12-hour) shifts. Because working inside those protective clothes with that mask in those conditions, it was taking all the energy of the staff and was boring, so we reduced their working hours so that they could work more efficiently. Participant No. 2.”*

In hospitals that admitted both COVID and non-COVID patients, to reduce the risk of staff infection, managers in the emergency department and triage of the hospital performed space separation and designated a dedicated nurse for COVID patients in each shift. At the same time, by assigning a code to patients with suspicious respiratory symptoms, they informed other personnel about the possibility of infection transmission from the very beginning of the patient’s admission.

*“We isolated the physical spaces. We separated the main triage from suspicious patients and set up a respiratory triage. We placed respiratory isolation where it was necessary so that patients could not enter the emergency room. Participant No. 8.”*

Other measures taken by the nursing managers during this period to prevent the infection of personnel were nutrition management, such as the distribution of snacks in the form of mineral water, fresh fruits and juices, and a variety of drinks, which equipped the pavilion in this regard. Furthermore, in order to maintain the energy of nurses and reduce their exposure to patients, arrangements were made to make all work equipment available.

In the pavilion, rest, and dining rooms, in addition to separating the space, measures such as disinfection, proper ventilation, detergents, and masks were made available. Also, disposable bed sheets were made available to the staff. In case of infection of nurses, to prevent the spread of disease to others, a special place for quarantine was considered.

#### Motivational measures

One of the most important issues that nursing managers mentioned for motivating the staff was the strong presence of managers, officials, and veteran experts as a model in the field. Many of them, especially in the first months of the outbreak, visited the wards and stayed there not only at the morning shift, but also evening shift, and some hours of night shift as well as holidays. They also carried out some follow-ups outside the hospital.

*“I think we should start with the authorities. Since the authorities themselves were at work in the center of the field, staff became motivated to work. Many officials visited the COVID wards and didn’t separate themselves from us. Participant No. 12.”*

Nursing managers in this study acknowledged that seeing the fatigue of nurses in this situation made them understand the staff more than before and have more cooperation with them. They did their management duties by the current situation and had more flexibility with their staff. They argued that in this situation, leadership alongside management has a better effect. In this crisis, a close relationship was established between the officials and the employees, and officials increasingly felt responsible and accountable.

*“When managers give orders from a distance, no one likes to obey the command and listens. In such situations, management is leadership. You have to be with the staff yourself, not just say a slogan and make staff feel that you just want to give them a command. Participant No. 3.”*

One of the measures taken by managers to increase motivation for nursing staff was the distribution of corona allowance among the staff working in COVID wards, and also the fair distribution of donations by public, charities, and public and private institutions staff. In some cases, the donations included flowers and gift cards. In addition to financial incentives, conveying a sense of appreciation to employees was an important motivating factor.

Timely payment of financial rewards is one of the factors that can act as a motivating factor for nurses’ intrinsic motivation and makes the difficult working conditions with corona patients more bearable for them.

Reducing working hours, giving per case allowance, reducing the number of shifts, and providing the opportunity for more rest between shifts, in addition to reducing the risk of infection, were also very important motivational factors. Since in many areas, nurses were also providing services during the New Year holidays, the managers promised them to leave after the number of patients decreased. Also, every opportunity was used to give a few days off and even a 10-day leave to staff for recovery.

*“For the New Year holiday, we convinced everyone that we do not have a holiday this year and everyone accepted, but we promised them a holiday after the end of corona lockdown. We told staff they can have 7-10 days off after the end of quarantine, while we disinfect the hospital. Participant No. 1.”*

Some temporary contract nurses were also highly motivated to work in the COVID wards for the possibility of future employment.

*“Some felt that their contract would change to a permanent one. Most* temporary contract *nurses were drawn to us in the hope that it would help them to be hired. Participant No. 6.”*

### Psychological support

In order to manage stress, in addition to providing psychological support, education, and calming anxious personnel, the managers provided the possibility of relocation for the staff and did not force them to work in the COVID wards. Nurses were also worked in rotation in the COVID wards. In the wards where their supervisors and managers were present, staff did not leave the service and were present during non-duty hours despite having problems such as a young child or elderly parents at home. In these wards, the nurses were better able to overcome their fears.

*“Yes, these are the first days, the staff was agitated. Then I told them, if you do not want to work in the COVID wards, it is not a problem at all. Anyone who does not want to work in the COVID unit for any reason come to me and write a request, and I do my best to send him or her to other units. Participant No. 3.”*

Nursing managers provided face-to-face or virtual psychological counseling for some staff and patients due to stress and anxiety.

*“We even took our psychologists to the wards to talk to staff and patients. We brought in a psychologist as a resident. Participant No. 6.”*

Other activities related to stress management that was used by managers included playing happy songs every morning in the ward, performing live concerts by famous radio and television singers in the hospital compound, paying homage by the military in the hospital, creating a warm and friendly atmosphere in the ward, and surprising the staff by hospital officials at a birthday party. As some nurses lived away from family members to reduce the risk of cross-contamination, authorities tried to create a friendly and family-like atmosphere in the ward.

*“For example, a very good job that our hospital did in the last two or three months was that they held a birthday party for every staff working in COVID wards, bringing a birthday cake, a present, and filming and congratulating the colleague on his/her birthday. Participant No. 9.”*

*“I am a head nurse and at work, I do not let the staff have a hard time. With a joke, I try to make them laugh and even between shifts, I make a drink for them myself, and I tell them they should have a drink. Participant No.14”.*

## Discussion

The findings of this study showed that nursing managers perceived management in the COVID-19 crisis differently from other crises. In their opinion, management in these difficult conditions is very complex and requires more flexibility of managers, and also should be based on the situation. Since the problem of shortage of nurses is an important challenge for health care systems around the world [24, 25] and Iran also faces this important challenge [26], according to the participants, the shortage in this crisis was exacerbated for various reasons included the high volume of work that COVID patients require, the difficulty of working with PPE and the exposure avoidance, and reduction in staff’s working hours due to the high risk of infection among hospital staff. Due to the concurrency of COVID-19 outbreak with the New Year holidays and Nowruz in Iran, and therefore the closure of many clinics and elective surgeries, and also because of reduced accident rates following home quarantine, reduced referrals of chronic patients for fear of infection in the hospital setting and the official announcement by the government in many provinces to ban cosmetic or non-emergency surgeries, the admission of patients in hospitals was reduced to even one-third of normal rate and this led to the closure of some wards. These factors greatly contributed to managing the great challenge of workforce shortages but were not enough. Nursing managers in the COVID-19 crisis made up for this shortage of staff by recruiting and using volunteers from other wards and hospitals and employing temporary staff. In an emergency, the WHO recommends the use of additional staff, including skilled personnel who can provide intensive care. Retired hospital staff, university staff, and students of medical, nursing, and public health universities are potential sources of additional staff [17]. However, participants in the present study did not mention the use of nursing students in caring for patients. In the fight against the COVID-19 pandemic, nursing managers in Taiwan recruited nursing staff to compensate for shortages of the workforce [27].

In this crisis, the shortage of nurses with skills and experience in working in the ICU is more prominent than anything else. In this regard, some studies have suggested that the government should empower nurses to learn the skills of working in ICUs and increase the number of ICU nurses at its own expense [24].

Since this disease is more common in older people with underlying diseases and debilitating problems, and because patient’s visitors are forbidden to enter the ward due to the spread of infection, patients needed help with their daily activities, including meeting their basic needs. Therefore, in addition to medical care, nurses also provided primary care, which in turn increases the workload of nurses. The lack of auxiliary workers was one of the major challenges for nursing managers, which in turn increased the workload of nursing staff and led to work burnout. One of the management strategies was to employ non-specialized volunteer forces for this purpose. Negarandeh et al. (2015) in their study suggested that one of the solutions to manage the shortage of nursing staff in the normal situation is to employ non-specialist nurse assistance workers [25]. According to studies, increasing workload and shortage of workforce have been mentioned as the most important limiting factors in controlling the spread of infectious diseases. Limited workforce and increasing the number of patients have significantly increased the workload of each nurse in the COVID-19 crisis and these factors can be detrimental to the physical and mental health of nurses [26]. The results of a study in the United States showed that with the adding of each patient and the increase in the workload of nurses, the probability of burnout and job dissatisfaction increases by 23 and 15%, respectively [28].

In addition to the measures taken to manage the staff shortage, changes were also made to the workforce arrangement during the pandemic. One of these important measures was the combination use of inexperienced and experienced personnel in shifts due to the arrival of a large number of recruits. In this regard, the combination of novice and experienced workforce together in the shifts emerged as one of the main categories in the study of Gao et al. (2020) during the COVID-19 outbreak, also nursing managers should reasonably evaluate the competencies and work abilities of nurses before setting shifts [29]. Additional staff, including volunteers, must also be approved for specific tasks, in terms of individual competence, responsibility, on-the-job training, and disease monitoring and prevention [17].

Providing the possibility of relocation for nurses with problems was another management strategy used for staffing in this study. Numerous studies have emphasized the need to support nurses and their families, and to ensure their safety in a variety of ways, including the provision of PPE, medications, and vaccines during the fight against a variety of infectious diseases and epidemics [30–35].

Nurses are under pressure not only because of the large number of patients but also because they are at risk of infection [27]. These factors lead to a shortage of nursing staff, which is one of the most important factors limiting the ability of hospitals to cope with the spread of emerging infections [36]. Therefore, in this study, nursing managers took measures to keep nurses in these wards and continue to serve COVID-19 patients to reduce staff infection, increase work motivation, and stress management in nurses.

During the COVID-19 pandemic, hospital officials in China by taking measures such as; providing intensive training, psychological counseling, as well as setting shifts rationally and scientifically, and putting emphasis on avoiding unnecessary contact, reduced the nurses’ infection and controlled their stress. In this study, to avoid unnecessary contacts to minimize disease transmission, hospitals were equipped with information systems, personal digital assistant systems, and local intranets, so all medical documents were paperless, and also nurses and doctors in separate rooms were able to assist patients remotely [37]. To prevent further infection among staff, it is necessary to set up a nursing shift program correctly using the available and efficient workforce resources [29].

One of the items that the participants in the present study mentioned as an important motivational factor were the strong presence of managers in the wards and their close relationship with the staff. In another study, managers’ closer relationship with nurses to increase motivation was mentioned as an important factor [29].

Due to the increase in psychological stress among employees, which can affect the proper provision of care, participants stated that the personnel’s stress can be reduced in various ways, including dialogue and close communication with employees and providing the possibility of psychological and psychiatric counseling. Studies emphasize the emotional support and protection of employees and their families, even emotional control such as fear and anxiety, and training on how to use PPE to encourage employees to be effective in their workplace [34, 35, 38].

The present study was conducted with a qualitative approach in which, a limited number of participants were purposefully selected. Therefore, its results cannot be generalized to the whole community of nursing managers. Due to the possibility of infection through contamination, it was not possible to conduct face-to-face interviews with all participants. Therefore, it was difficult to get the non-verbal reactions of the participants. Also, the level of respondents’ trust in the interviewer could not be determined. So, the researcher tried to explain the purpose of the interview and ensure the participants that their personal information will remain completely confidential and this information will be used only scientifically to gain the participants’ trust as much as possible.

## Conclusion

In this study, conventional content analysis was used. The interviews revealed the nursing managers’ perceptions of nursing staff management in hospitals involved in caring for COVID patients. Management in an unwanted and prolonged crisis like the COVID-19 pandemic is very difficult and different from other crises and maneuvers. We found that the management of nursing staff in this crisis included the appropriate recruitment, employment, replacement, and relocation of staff. Besides, to increase the quality of nursing care and patient safety, it is necessary to first examine the newcomers in terms of scientific and practical competencies and capabilities, and then place them in combination with other staff in different departments appropriately and efficiently. This staff is faced with many challenges, so managers should take measures to increase their motivation and reduce their risk of infection and stress. In this crisis, managers dynamically used the principles of situational management and flexibility. Nursing managers with a strong presence in the field, despite having problems, were a good role model for other staff.

## Supplementary Information

**Additional file 1.** Interview Guide

## Data Availability

Data are available by contacting the corresponding author.

## Abbreviations

COVID-19: Coronavirus Disease 2019
ICU: Intensive care unit
MERS-CoV: Middle East respiratory syndrome coronavirus
WHO: World Health Organization
SARS: Severe Acute Respiratory Syndrome
TUMS: Tehran University of Medical Sciences
PPE: Personal protective equipment
