# Supplementary material for: Exploring nursing managers’ perceptions of nursing workforce management during the outbreak of COVID-19: a content analysis study
Source: BMC Nurs. 2021 Jan 29;20:27. doi: 10.1186/s12912-021-00546-x (PMC7844784; doi:10.1186/s12912-021-00546-x)
Supplement: Supplementary file 1 — Additional file 1. Interview Guide [file 12912_2021_546_MOESM1_ESM.docx]

**Additional file: Interview Guide**

| **Box 1. Semi-structured interview guide**  What is your understanding of the management of nursing staff in the hospital setting during the outbreak of COVID-19?  What challenges have you faced in managing the nursing staff during the COVID-19 outbreak?  What strategies do you use to manage the nursing staff?  Can you give an example of this? Or "Can you explain more about this?  Is there anything else you would like to explain? |
| --- |
